# Sense of belonging and its positive association with physical activity levels and negative association with sedentary behaviors in residential aged care facilities in COVID-19 pandemic: a longitudinal study

**DOI:** 10.3389/fpsyg.2025.1529463

**Published:** 2025-02-05

**Authors:** Gonzalo Marchant, Emma Guillet-Descas, Natacha Heutte

**Affiliations:** ^1^Center for the Study and the Transformation of Physical Activities (CETAPS) UR 3832, University of Rouen Normandy, Mont-Saint-Aignan, Normandy, France; ^2^Laboratory L-ViS, F-69622, Université Claude Bernard Lyon 1, Villeurbanne, France

**Keywords:** relatedness, older adults, SARS-CoV-2, behaviors, repeated measures analyses, correlations

## Abstract

**Introduction:**

The COVID-19 pandemic reduced physical activity levels and increased sedentary behavior among older adults in residential care facilities. Another effect of this crisis was that facilitating a sense of social belonging through in-person social activities, such as group exercises or communal meals, became difficult. This study examines the relationship between physical activity, sedentary behavior, and sense of social belonging in older adults.

**Methods:**

This longitudinal study, which lasted 10 months, involved 57 older adults in residential care facilities. Participants completed the *Échelle de mesure du Sentiment d’Appartenance Sociale* (*ESAS*) questionnaire three times and wore an accelerometer on their waists for 1 week each time to measure sedentary time and physical activity levels. The *ESAS* questionnaire, a validated tool for evaluating social belonging in older adults, measures social belonging through a series of questions that assess an individual’s feelings of acceptance and intimacy within their social group.

**Results:**

Participants were predominantly sedentary (7.30 h/day) and engaged in low-intensity physical activities (2.9 h/day). They did not meet the recommended 150 min/week of moderate-to-vigorous physical activity. The sense of social belonging was high, with mean scores of 6.75 (*ESAS*), 7.08 (Acceptance), and 6.43 (Intimacy). Light physical activity was negatively associated with sedentary time. The sense of social belonging was positively associated with light physical activity and negatively correlated to sedentary time.

**Conclusion:**

This study underscores the importance of light physical activity and a sense of social belonging in reducing sedentary behavior among older adults in residential care facilities. By promoting social interactions and light physical activity, we can enhance the well-being of this population, especially during pandemic conditions. The study’s findings should inspire future interventions to focus on these aspects, thereby improving health outcomes in residential care facilities for older adults.

## 1 Introduction

### 1.1 Effects of physical activity and sedentary behavior on older adults’ health

Ageing is a natural life stage involving various physical, psychological, and social changes. As people age, it is important to adopt measures to maintain or improve their quality of life to age actively and healthily. Among the most recommended non-pharmacological strategies to improve quality of life in old age is the practice of physical exercise (Parra-Rizo, 2020). However, older adults have been described as inactive people with low physical activity (PA) levels (Sun et al., 2013) and high sedentary behavior (SB) levels (Bellettiere et al., 2015; Harvey et al., 2015). One consequence of inactivity in older adults is poor physical performance and the risk of increasing their dependence on activities of daily living (Roberts et al., 2017). In addition, this may reduce the perception of quality of life and general health (Cunningham et al., 2020). On the contrary, when older adults engage in PA, they have a positive perceived health-related quality of life (Yen and Lin, 2018). In terms of SB, a systematic review of the literature on this behavior and the health outcomes among older adults showed that greater SB time was related to an increased risk of all-cause mortality (Machado et al., 2014).

### 1.2 Living environment and older adults’ behaviors

The potential impact of the living environment on PA and SB in older adults, particularly in residential care facilities (RCFs), is a topic of significant relevance and interest. Older adults in nursing homes tend to decrease their PA levels (den Ouden et al., 2015) and increase their SB even more than older adults living independently in community or assisted-living facilities (Ustad et al., 2024). Older adults’ activities in RCFs are primarily sedentary (79%), engage in low-intensity PA (20%), and there is almost no moderate-to-vigorous (1%) physical activities (Barber et al., 2015). Furthermore, the use of objective PA measures has uncovered a significant finding: light physical activity (LPA), defined as one to three metabolic equivalent task (MET) hours (e.g., light walking), is inversely associated with SB and positively linked to well-being in older adults (Buman et al., 2010). Therefore, validated moderate-to-vigorous intensity thresholds of PA for health (i.e., 5 METs to 20 METs and above) in general adult populations may be high for older adults. These thresholds must fail to capture the important associations between LPA and health in older adults. Studies have also shown that older adults have difficulty self-assessing their LPA levels (Washburn, 2000). In Ustad and colleagues’ study (2024), older adults had lower levels of PA by type of care attention. Thus, older adults who received higher care attention stood less often and walked less. In the same study, standing was the dominant type of PA and walking appeared predominantly in short periods at all levels of care. For this reason, LPA constitutes the main component of daily PA in older adults, which argues for a more precise study of the role of these activities in the maintenance of daily PA and health at an advanced age (Ustad et al., 2024).

### 1.3 Impact of the COVID-19 pandemic on older adults

During the COVID-19 pandemic, the French government restricted all non-essential internal movement -defined as a lockdown- on three occasions (from March 17 to May 11, 2020; from October 30 to December 15, 2020; and from April 3 to May 3, 2021) to curb the spread of coronavirus 2019. This health measure included physical distance, restricted access to residential care facilities, and social isolation of older adults living in RCFs. The closure of the RCFs for older adults was part of a series of policies restricting human contact and travel in response to the pandemic (for an average of 22 days in a lockdown situation). This measure had adverse effects on older adult’s health, including psychiatric and medical comorbidities (Roy et al., 2023). An additional consequence of the lockdown was the reduction of PA levels in older adults (Lebrasseur et al., 2021) and SB increase (Caruso Soares et al., 2022; Hahn et al., 2023).

### 1.4 Sense of social belonging: its importance and relationship with physical activity and sedentary behavior in older adults

One factor that could positively affect PA levels is the Sense of Social Belonging (SSB). Older adults with high levels of SSB tend to engage in more PA (Lindsay Smith et al., 2017). The World Health Organization (WHO) also identifies SSB as a critical determinant of Active ageing (World Health Organization, 2002). The SSB was a fundamental human need disrupted by social distancing applied to prevent the spread of the SARS-CoV-2 virus (Derrer-Merk et al., 2022). This condition also led to isolation and, in some cases, an increased sense of loneliness in older adults (Gardiner et al., 2020). According to Allen et al. (2021), the SSB refers to the need to belong based on experiences of social interactions, while loneliness is a subjective feeling of having less social contact than one wants. The SSB considers the intimacy and closeness between two or more people. It also includes a feeling of acceptance, meaning that the individual feels understood and listened to by people they trust and who mean something to them (Richer and Vallerand, 1998). Moreover, SSB appeared to protect against loneliness at home and in RCFs in older adults (Prieto-Flores et al., 2011). While the COVID-19 crisis highlighted the importance of these factors, diminished SSB and loneliness are conditions that existed prior to this pandemic in older adults living in RCFs. Several life events could explain social distancing and isolation in older adults; for example, retirement, widowhood, health problems, divorce (Sheftel et al., 2024), or moving into RCFs or nursing homes (Boamah et al., 2021). It is clear that social distancing and loneliness are significant health risk factors for older adults (Lapane et al., 2022; Valtorta et al., 2016).

#### 1.4.1 Sense of social belonging and sedentary behavior

In the context of SB, several studies have shown that social interactions and social capital can influence the time spent in SB. However, this relationship depends on the type of SB. Studies have shown that passive mental SB such as television viewing are often associated with decreased social interaction. For example, time spent watching television can reduce the amount of time spent on activities with neighbors, which can lead to a decrease in SSB and, consequently, a decline in emotional well-being (Yasunaga et al., 2021). Conversely, more mentally active SB, such as chatting with others or participating in leisure activities, are often associated with increased social interactions. These interactions can reinforce SSB and contribute to a more significant state of happiness and life satisfaction (Yasunaga et al., 2021). Social capital, which encompasses social networks and norms of trust and reciprocity within a community, plays a crucial role in mediating between SB and happiness. Studies have shown that participating in activities with neighbors can mediate between SB and happiness by fostering social interactions that reinforce belonging (Yasunaga et al., 2021). The effects of SSB on SB can vary between adults and older adults, with older adults tending to spend more time in passive SB, which can affect their social capital and well-being (Anderson et al., 2016; Russell and Chase, 2019). Conversely, the SSB in older adults living in RCFs could be an essential risk factor for the protection of health. Older adults with higher levels of SSB have better quality of life and reduced depression (Lim et al., 2023). Adults with a stronger sense of community belonging were more likely to report low SB time during leisure (Anderson et al., 2016). Otherwise, loneliness seems unrelated to SB measured with accelerometers (Schrempft et al., 2019). However, men and women aged 50–81 who reported not being socially isolated reported lower SB and higher time spent participating in moderate- to vigorous-intensity PA than those who reported being socially isolated (Schrempft et al., 2019).

Most studies of PA and SB in older adults in RCFs have used questionnaires (D'Amore et al., 2023). The main limitation of this method is that older adults would overestimate their PA levels and underestimate SB levels compared to accelerometer measures (Dohrn et al., 2020). Likewise, research on device-based (i.e., accelerometers) PA and SB in older adults is scarce (Aaltonen et al., 2023). Furthermore, most studies assessing this population’s PA levels and SB are cross-sectional (D'Amore et al., 2023). Recent studies indicate that it is necessary to study these factors long-term to understand further the extent to which SSB impacts health outcomes in older adults in congregate living situations (Lim et al., 2023). Thus, our study aims to fill the gap in PA and SB assessment using objective measures (accelerometers), specifically analyzing light-intensity activities in older adults. In addition, we will study the relationships that may exist between SB, PA levels and the SSB of older adults living in RCFs. Our approach will be longitudinal as most of the studies that have studied SB and PA levels and their relationship with SSB are cross-sectional studies. Finally, we will propose practical aspects of the application of our results.

### 1.5 Objectives

The objectives of this study are to complement previous research and address specific knowledge gaps by:

- Utilizing accelerometers to provide more accurate and objective measurements of Physical Activity and Sedentary Behavior in natural settings, addressing the limitations of self-reported data.
- Conducting a longitudinal study to understand the long-term relationship between Physical Activity, Sedentary Behavior, and Sense of Social Belonging of older adults in Residential Care Facilities.

### 1.6 Hypothesis

1. Older adults living in residential care facilities would spend less than 150 min per week on moderate-to-vigorous physical activity, and their main physical activity would be light intensity.
2. Older adults would spend most of their waking time sedentary.
3. Light physical activity levels would be negatively associated with sedentary behavior.
4. Light physical activity levels will be positively associated with the sense of social belonging.
5. Sedentary behavior time will be negatively associated with the sense of social belonging.

## 2 Methods

### 2.1 Participants

In total, 54 older adults (49 women and five men) living in residential care facilities (RCFs) participated in this project. The older adults had lived, on average, 5 years and more in the residences, and most of them participated in a Physical Activity (PA) program in their residences. Only 27 persons validated the accelerometer’s data and completed the Sense of Social Belonging (SSB) questionnaire over the three measures (Table 1). In the first accelerometer measurement, 27 people did not want to wear the device. In the second measurement with the accelerometer, seven people forgot to wear the devices and decided not to continue in the study. Three people were absent for the third measurement time because they were transferred from their residence to a nursing home without returning.

**Table 1:** Descriptive statistics.

	Mean (sd) or *n* (%)			
Demographics				
Age	83.46 (*sd* = 8.21)			
Gender				
Women	49 (90%)			
Men	5 (10%)			
Years living in residence	5.78 (*sd* = 5.66)			
Physical activity participation				
Yes	66%			
No	34%			

**Behaviors**	Measure 1	Measure 2	Measure 3
	*n* = 27	*n* = 20	*n* = 17
SB (mean minutes of 5 days)	2,184.71 (265.32)	2,183.89 (340.36)	2,209.79 (299.03)
LLPA (mean minutes of 5 days)	329.11 (100.92)	327.83 (105.64)	339.56 (117.18)
LHPA (mean minutes of 5 days)	486.180 (193.72)	488.27 (254.85)	450.64 (202.21)
MVPA (mean minutes of 5 days)	126.159 (149.44)	133.562 (170.24)	93.219 (101.17)

**Sense of social belonging**	*n* = 27	*n* = 23	*n* = 21
*ESAS* score	6.75 (1.83)	6.76 (2.19)	6.74 (1.86)

SB, sedentary behavior; LLPA, low light physical activity; LHPA, low high physical activity; MVPA, moderate-to-vigorous physical activity; ESAS, sense of social belonging scale.

#### 2.1.1 Recruitment

This study’s recruitment process included posters inviting adults living in residential care homes to a study briefing meeting (60 min) in Lyon City, France. During the meeting, there was a presentation about the objectives of the study. At the end of the meeting, 73 people expressed their interest in participating in the study. Then, individuals were presented the details of the study, the letter of invitation (Supplementary material S1), and the consent form (Supplementary material S2).

#### 2.1.2 Inclusion and exclusion criteria

**Inclusion:** Adults living in residential care facilities and autonomous in their daily activities residing in Lyon city who attended the project information meeting were included.

**Exclusion:** People with pacemakers, mobility problems, persons unable to consent, and people who planned to change establishments within the 12 months following the interview were excluded.

### 2.2 Tests administered

#### 2.2.1 Questionnaires on demographics and sense of social belonging

The questionnaire was structured with relevance in mind, divided into two parts in paper format. The first part focused on the participants’ demographics, while the second part explored their Sense of Social Belonging. The details of this relevantly structured questionnaire are presented as follows.

##### 2.2.1.1 Demographics

This questionnaire was about participants’ gender, age, time (months/years) living in a facility care residence, and physical activity program participation in the residence.

##### 2.2.1.2 Sense of social belonging

The questionnaire of the Sense of Social Belonging (*Échelle de mesure du Sentiment d’Appartenance Sociale: ESAS*; Richer and Vallerand, 1998) involves two dimensions: acceptance and intimacy. Acceptance refers to the feeling of being understood, accepted, and respected. Intimacy refers to closeness or emotional attachment with other individuals. The statement was: ‘In my relationships with my co-residents in this house, I feel…’. Acceptance item example:… “in confidence with them.” Intimacy item example: … “estimated.” There were five items for each dimension on a 10-point scale where “1 = Strongly disagree” to “10 = Strongly agree.”

#### 2.2.2 Physical activity and sedentary behavior measures

Physical activity and sedentary behavior were measured using Actigraph® GT3X accelerometers programmed to record activity in 60-s epochs. Participants wore the device on the hip thrice for 7 days, only removing it for bathing, swimming, and sleeping. To analyze data from the accelerometer, participants had to wear it for at least four-week days and one weekend day, and for at least 10 h/day of valid wear time (Curry and Thompson, 2014).

### 2.3 Procedure

**Figure 1:**
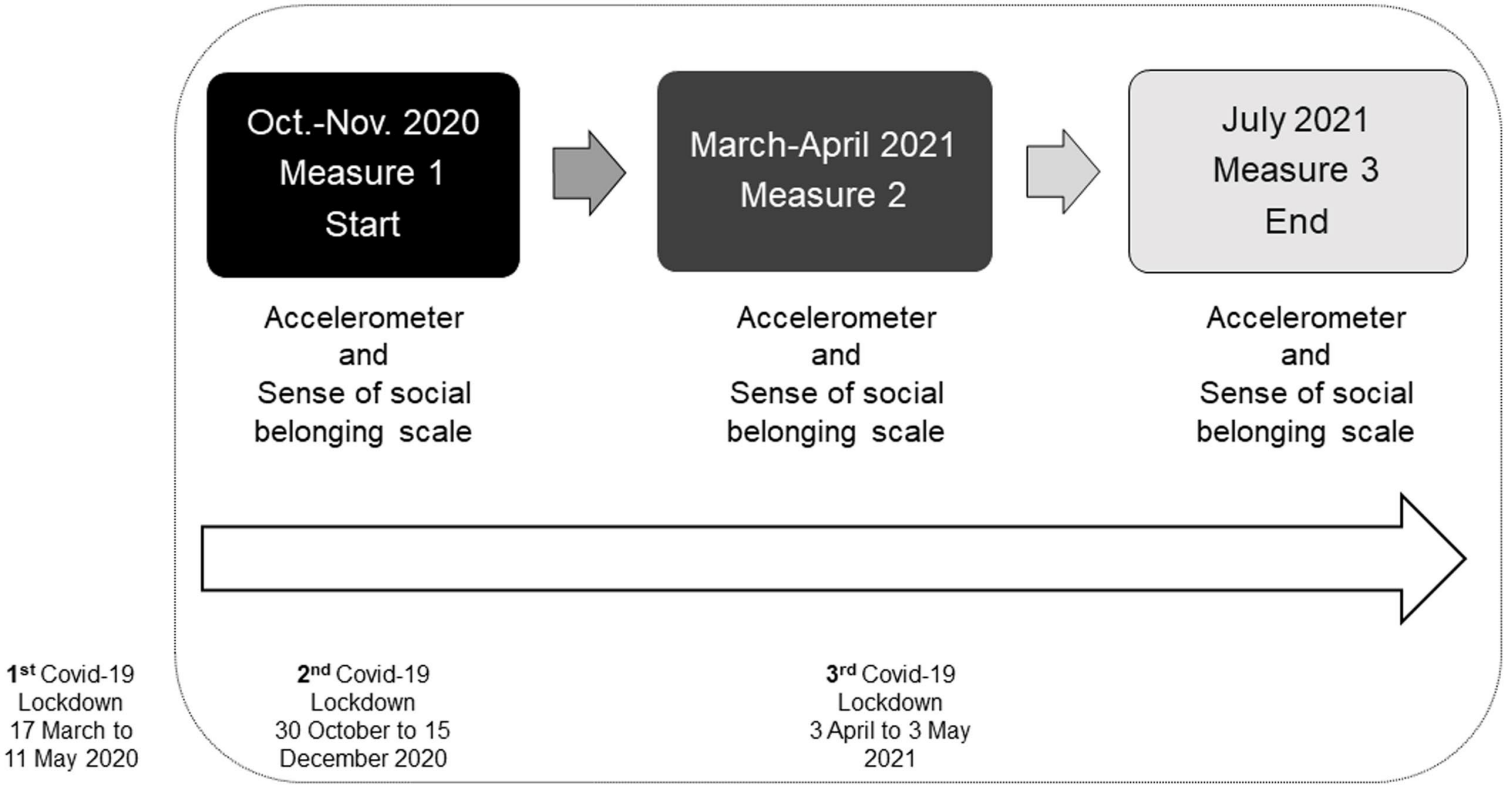
Study timeline.

From October 2020 to July 2021, 57 subjects underwent a multidimensional assessment. There were three assessment times over 10 months for the sense of social belonging, physical activity, and sedentary behavior (Figure 1). The first measurement time, which included questionnaires and accelerometers, was from 2 October 2020 to 6 November 2020. These questionnaires were comprehensive, including a demographic part to ensure a thorough understanding of the participants. The participants began wearing the accelerometers 1 day after completing the questionnaires. The second measurement time, which focused on the sense of social belonging questionnaire and the accelerometers, was from 20 March 2021 to 25 April 2021. The third evaluation, which was identical to the second one, was from 29 June 2021 to 30 July 2021. The mean delay between the administration of the tests and measurements with the accelerometers was 39 days (*SD* = 17.20). This delay was due to the time that had to be waited for the quarantine of some participants who contracted the Covid-19 virus.

### 2.4 Ethical considerations for data collection during lockdowns

The study was conducted according to the guidelines of the Declaration of Helsinki and was consistent with the ethical principles specified in the APA standards. Participants who were asked to participate in the study were given details of their required involvement, and they were assured their right to withdraw. They were also provided with a consent form describing the study’s aim and procedure and gave their written consent. Standard verbal and written instructions regarding the content of the questionnaires were then provided. Instructions emphasized the confidentiality of individual responses and the need for honesty. The participants were assured that the results would be used only for this study and that their privacy would be guaranteed.

The ethical considerations for data collection during lockdowns included proximity and contact tracing (StopCovid®France), symptom monitoring (person in charge of the residence), and quarantine control (Gasser et al., 2020). Research assistants should not have had Covid virus in the last 14 days. If this was the case, the person transporting the devices was changed. Two days before going to the residences, the research assistants carrying the accelerometers had to take an anti-virus test. The accelerometers were deposited outside the residence in a dedicated box if the test was negative. If a participant was in quarantine, the accelerometers were rescheduled for 7 days later. It was also verified that all the people who participated in our study were up to date with their vaccinations.

#### 2.4.1 Adaptations to conduct the study during the COVID-19 pandemic

The research team established strict health safety measures to conduct the study under pandemic conditions. The first measure was to set up a relay with the staff of residential care facilities for the older adults. The research team sent the questionnaires by e-mail to the managers of the people in the residences. These questionnaires were then printed out. The accelerometers were cleaned before they were handed over to the people in charge of the residences. Then, the team of the residences administered the questionnaires (with anonymous codes) and was in charge of delivering and collecting the accelerometers (with individual and anonymous codes). Each participant had a code (EA123) which ensured his anonymity. The research team then obtained authorization from the directors of the residences to collect the questionnaires and the accelerometers. Finally, each device was cleaned with special products to prevent the transfer of any viruses or bacteria to the adult residents. This process was carried out systematically before and after each evaluation time.

### 2.5 Statistical procedures

The data from the questionnaires were entered in Excel files. Then, the codes of the questionnaires were paired with those of the accelerometers. Data from accelerometers (GT3X) were cleaned and scored using ActiLife software (version v6.13.4). There were 90 consecutive minutes of zero counts defined as Non-wear time, with an allowance of 2 min of nonzero counts provided there were 30-min consecutive zero count windows up and downstream (Choi et al., 2011). Participants should wear the accelerometer at least 10 h per day with 4-week days and 1-week end day. There were cut-off points for Sedentary time (<100 counts/min) and Moderate-to-Vigorous Physical Activity (≥1,952 counts/min). There were two categories for defining Light Physical Activity. The Copeland and Esliger threshold (2009) distinguishes between Low-Light Physical Activity (LLPA; 100–1,040 counts/min) and High-Light Physical Activity (HLPA; 1,041–1,951 counts/min). We used the GT3X+ low-frequency extension option (GT3X + LFE), which increases sensitivity to very low amplitude activities and is suited for older adults who may move slowly or take very light steps for all accelerometer data (Cain et al., 2013).

Descriptive statistics include the means, the standard deviations (*SD*), or the proportions (n, %) for continuous and categorical variables. A repeated measures correlation (Bakdash and Marusich, 2017) was performed for the association between PA, SB, and SSB over three times of measures. Statistical analyses were performed using SAS statistical software version 9.4.

## 3 Results

Age was a factor related to levels of sedentary behavior and physical activity. The older the age, the more sedentary time (*r* = −0.425, *p* = 0.026) and the less time in High-Light Physical Activity (*r* = −0.414, *p* = 0.031).

### 3.1 Sedentary and physical activity levels

Older adults living in RCFs were mainly sedentary (7.30 h/day) and inactive (<150 min of Moderate-to-Vigorous PA/week). Their PA was low intensity, almost 3 h per day (*M* = 2.9, *SD* = 1.02).

### 3.2 Sense of social belonging (*ESAS*)

Older adults living in RCFs had a high perception of SSB. The scores of Acceptance and Intimacy were, on average, high, with a mean of 7.08 points (*SD* = 1.96, 95% *CI* [6.61, 7.54]) and 6.43 (*SD* = 2.05, 95% *CI* [5.95, 6.91]), respectively. *The ESAS* score was high, with a mean of 6.75 points (*SD* = 1.94, 95% *CI* [6.30, 7.21]).

### 3.3 Association between sedentary, physical activity levels, and sense of social belonging over the three times of measure

**Figure 2:**
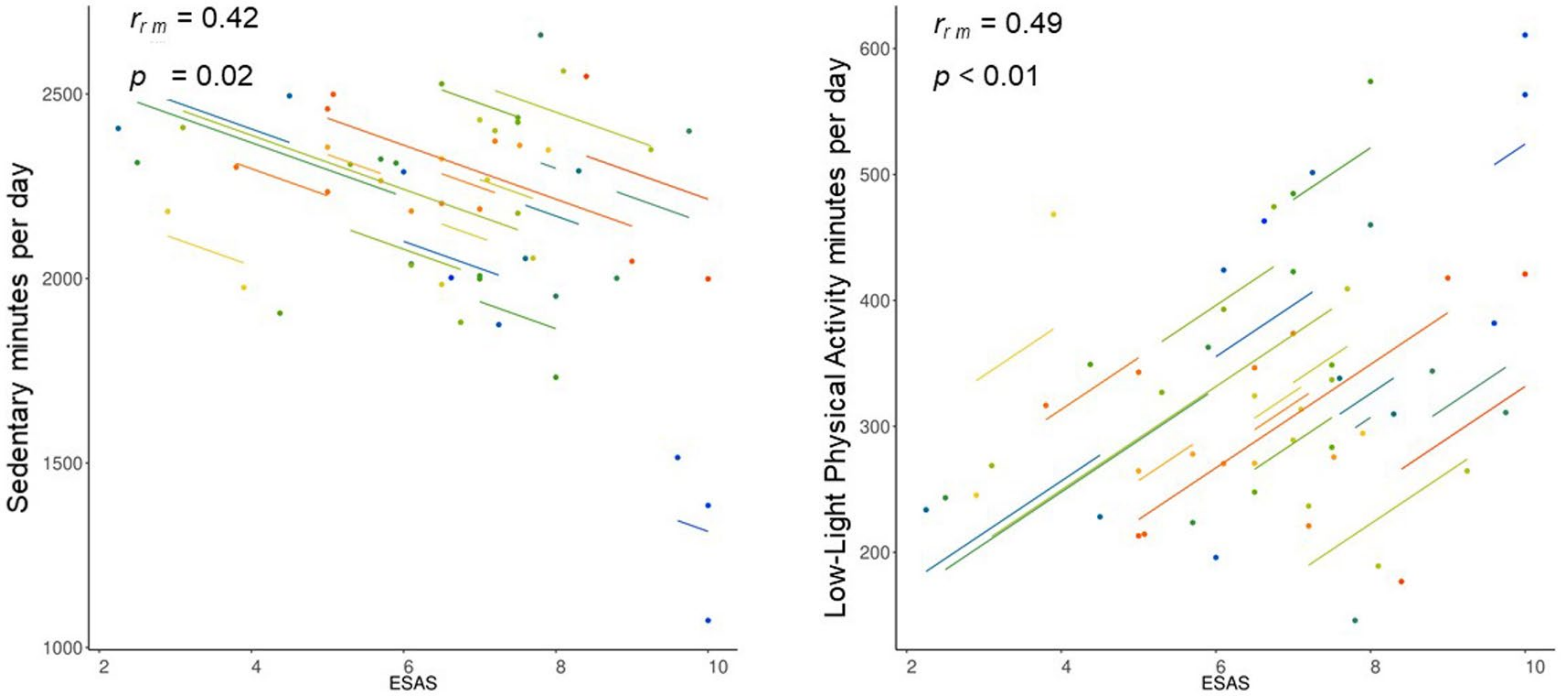
Repeated measures correlation of sedentary and low-light physical activity with sense of social belonging (ESAS).

Table 2 shows repeated measures correlations results over the study. Sedentary time was significantly and negatively related to Light PA intensities (i.e., Low-Light PA and High-Light PA) and the ESAS (Figure 2).

**Table 2:** Correlations of sedentary time, physical activity and sense of social belonging constructs over 10 months.

	2. LLPA	3. LHPA	4. MVPA	5. *ESAS*
1. Sedentary time-min/week	−0.827, *p* < 0.0001	−0.916, *p p <* 0.0001	−0.211, *p* = 0.289	−0.422, *p* = 0.019
2. LLPA-min/week		0.532, *p =* 0.0005	0.095, *p* = 0.637	0.490, *p* = 0.005
3. LHPA-min/week			0.397, *p* = 0.039	0.286, *p* = 0.124
4. MVPA-min/week				0.004, *p* = 0.767
5. *ESAS*				1

LLPA, low light physical activity; LHPA, low high physical activity; MVPA, moderate-to-vigorous physical activity; ESAS, sense of social belonging.

Regarding the different PA intensities, there was a significant and positive relation between HLPA and Moderate-to-Vigorous PA levels. In addition, LLPA was significantly and positively associated with the ESAS dimensions over the 10-month study duration (Figure 2).

## 4 Discussion

Our study aimed to objectively assess the levels of sedentary behavior (SB) and physical activity (PA) of older adults living in Residential Care Facilities (RCFs) during a 10-month follow-up and determine the long-term relationship between SB, PA and the Sense of Social Belonging (SSB).

### 4.1 Physical activity levels of older adults living in residential care facilities

Our first hypothesis was that older adults living in RCFs would be inactive. Our results showed that older adults did not reach the recommended levels of PA for health. This result could have different adverse effects on the health of this population, such as increased sarcopenia due to physical inactivity (Sánchez-Sánchez et al., 2024). On the one hand, this result shows that older adults living in RCFs have low levels of PA (den Ouden et al., 2015).

The age and gender of the participants in our study emerged as crucial factors in understanding the observed low PA levels. Our study sample, predominantly comprising women over 80 years old living in RCFs, provided significant insights. Ustad et al. (2024) compared the daily PA of older adults based on care levels. Their findings revealed that older adults with a higher level of care exhibited lower daily PA, attributed to their advanced age and the higher representation of women in this group. Our study further confirmed that as individuals age, their PA levels tend to decrease.

Therefore, a nuanced analysis of our results within the specific French context of RCFs is essential. In France, the life expectancy of women is 85 years old and men 80 years old, and the entry in RCFs for women is 87 years and 82 years old for men. At older ages in France, women are much more likely than men to be in a situation of recognized dependency (i.e., in RCFs), which could translate into less movement and a consequent decrease in PA levels. The results of the National Institute of Statistics and Economic Studies (Perona, 2023) show that 14% of women receive personal independence allowance between the ages of 80 and 84, 28% between the ages of 85 and 89, and 55% after the age of 90, compared with 9, 18, and 41% of men, respectively. This condition could explain why more women would be in residential care homes for older adults (Hahn et al., 2023).

Gender, among French older adults, is an important discriminating factor in the PA performed. Regarding domestic activities, men practiced more gardening and do-it-yourself activities, whereas women were more involved in household activities (Pierre et al., 2022). Thus, when older women enter residential care homes, their levels of physical activity drop even further due to the nature of the care provided there. Thus, PA levels depend on the care regime (e.g., assistance with household chores such as cleaning or cooking and going out to do the shopping). This condition means that the more assistance they have, the less they move in this context, particularly if they are women. This result also indicates that there are cultural factors or differences in physical functioning between older men and women (Bellettiere et al., 2015).

During the COVID-19 period, older adults could leave their rooms and residences. They may have preferred to stay in their rooms or residences, limiting their PA even more and reducing the time spent in PA of light intensities. Similarly, the pandemic context and the restrictions in France, particularly the lockdowns, were sufficiently long (i.e., 22 days on average), strict (5 km movements around the primary residences), and each departure required a certificate with authorization to leave the residences. In these conditions, older adults likely did not have access to these documents either, as they had to be requested via the Internet, printed out, and completed. Several studies have shown that digital literacy among older adults is poor, and during the COVID-19 crisis, this problem became even more evident (Nedeljko et al., 2022).

Cultural context also played an important role in expressing PA. In France, there are two institutional support offers for older adults and their families. The first option is to stay at home with services, a choice made by most French (81%). The second option is to move to an accommodation structure for older adults, such as a medico-social institution or a foster family. However, frailty and institutionalization at the end of life often become unavoidable (Roy et al., 2018). It is important to understand that for older adults, moving into an institution is experienced as a form of care that presents more rules and an impersonal way of treating them (Chammem et al., 2021). In this sense, if the rules of the RCFs were restrictive and without major interventions to promote PA, older adults may have felt that they should not break the rules and stay inactive.

Some studies have suggested that European, United States (US), and Canadian cultures are more lenient towards physical activity compared to Asian cultures (Chen and Biswas, 2022), which tend to be more stringent (e.g., China, Singapore, South Korea, Japan). However, France’s adoption of strict measures such as lockdowns, social distancing, and mask-wearing, in contrast to countries like Sweden, which argued against lockdowns, adds a layer of complexity to the relationship between culture and physical activity. These cultural factors could significantly influence physical activity and sedentary behavior levels, thereby enriching the interpretation of our results.

Additionally, outdoor activities for older adults in RCFs are reduced because they often have difficulty getting outdoors independently (Nordin et al., 2024). For these reasons, the life context of older adults is a determinant of PA levels and SB (den Ouden et al., 2015; Ustad et al., 2024). Some studies have shown that leisure-time physical activity is the most reduced in older adults. Therefore, it would be interesting to know in detail if these activities exist in nursing homes and, above all, if they are motivating/attractive or not to participate in (Sun et al., 2013).

### 4.2 Sedentary behavior in the residential care facilities

We confirmed the second hypothesis of our project, as older adults spent more than half of their time in SB. Our result is consistent with the findings of Harvey et al. (2015), who reported more than 9 h of SB. Our study did not distinguish the types of SB. This condition is essential because it could indicate more precisely which activities and contexts lead older adults to spend their time sitting or lying down. On the other hand, several studies have indicated that the older adults get, the greater the fear of falling (Aaltonen et al., 2023), which would increase the time spent in SB but had no effect on the number of falls (Caruso Soares et al., 2022). Thus, other factors may influence the accumulation of older adults living in RCFs during this time (Bellettiere et al., 2015). For example, for men and women, a more significant proportion of time spent being sedentary took place during the later hours of the day than the earlier (Bellettiere et al., 2015), but women accumulated less sedentary time overall.

Another aspect that should be considered is older adults’ perception of SB. According to a systematic review by Compernolle et al. (2020), older adults consider physical inactivity synonymous with SB. In addition, older adults acknowledge that they do not know SB and the health risks associated with this behavior. Generally, older adults consider sitting part of their habits, mainly if sitting is associated with comfortable or enjoyable activities (Compernolle et al., 2020). Some studies indicate that spending more time sitting when older adults are in RCFs depends on the expectations of family or friends, who encourage older adults to sit more (Niklasson et al., 2023). In addition, being sedentary means for older adults a lack of social interactions associated with the ageing of the body and the physical limitations this entails. Therefore, in addition to the context of RCFs, motivation, family environment, and physical limitations of an ageing body should be considered when analyzing SB in older adults living in RCFs.

### 4.3 Association between physical activity and sedentary behavior in older adults

The third hypothesis, establishing a negative relationship between Light Physical Activity (LPA) levels and SB, was confirmed only for Low-Light PA. This result is because the Low-Light PA levels, which characterized this study’s population, reached 2 h per day. Several studies have shown that LPA positively affects older adults’ health (Ustad et al., 2024). Low-Light PA allows older adults to compensate for SB time through this intensity (Buman et al., 2010). Our results showed that the time spent in SB negatively correlated with light activities (i.e., Low-Light PA and High-Light PA). Another explanation of this result is that we are the first studying the relationship between two LPA thresholds (Copeland & Esliger2009) distinguishing between Low-Light Physical Activity (LLPA; 100–1,040 counts/min) and High-Light Physical Activity (HLPA; 1,041–1,951 counts/min). This condition, added to the low-frequency extension option for the accelerometer GT3X®, increased sensitivity to very low amplitude activities and is suited for older adults who may move slowly or take very light steps for all accelerometer data (Cain et al., 2013). Additionally, compared to previous transversal studies (Aaltonen et al., 2023), our longitudinal study showed that the negative association between SB and Low-Light Physical Activity could be maintained.

Our analyses only established a correlation; however, our results show that increasing these intensities in older adults could play a dual role in reducing SB and increasing PA levels. This condition is critical as every movement keeps older adults active (World Health Organization, 2002). According to Barber et al. (2015), older adults performed low PA intensity, such as transferring from a chair and self-care activities. In this way, low-intensity activities that are part of everyday life should be a focus of attention to promote PA in older adults living in these contexts. Concrete examples could be to increase transitions. One measure could be to encourage light transitional PA (i.e., a change from sitting to standing), leisure and recreational walking or dancing, and active transport (i.e., grocery shopping). Similarly, the positive relationship between High-Light PA and Moderate-to-Vigorous PA levels showed that promoting PA in older adults living in residential care should include progressivity as an essential principle. Low-intensity activities should be analyzed in a human movement continuum, not only for older adults. The relationship was positive and significant only between High-Light PA and Moderate-to-Vigorous PA. For this reason, the threshold of PA and SB could be determinants of the studies’ results. In our study, the Copeland and Esliger (2009) threshold distinguishes between Low-Light PA and High-Light PA, making our results more accurately reflect the intensities of an older adult population living in RCFs. These results have implications for equating PA recommendations for health, as expecting older adults in residential settings to move at moderate to high intensities would be like asking a 50 km/h car to accelerate at 95 km/h. Therefore, assessments should include a continuum from intensities associated with SB through different levels of light intensities and ending with moderate and vigorous activities. Therefore, assessing PA via accelerometers in older adults should use parameters sensitive to light intensities and avoid Troiano parameters in RCFs (Buman et al., 2010).

### 4.4 Physical activity levels and sense of social belonging associations

Regarding the fourth hypothesis, there was a long-term relationship between Low-Light PA and a sense of social belonging (SSB) in older adults living in RCFs. This result is significant as it indicates that, on the one hand, PA may play a role in physical health and psychological (i.e., loneliness) and social health as residential satisfaction (Prieto-Flores et al., 2011). On the other hand, it confirms that higher levels of SSB are associated with higher levels of PA over the 10 months of follow-up (Lindsay Smith et al., 2017). Similarly, the relationship between PA levels and the SSB was likely a protective factor for older adults’ health during the COVID-19 pandemic. In addition, this positive relationship continued during the 10 months of follow-up in the study. Thus, our study showed that a virtuous relationship between PA levels and SSB may exist in the long term. However, more studies are needed to establish the causal relationships between these two variables, as most studies have been observational and cross-sectional. The association between the SSB and the Low-Light PA results is logical considering the length of time people have lived in residence and the fact that Low-Light PA characterized PA in older adults. Most of the study participants lived in these residences for more than 5 years, which confirms that the SSB is a process that takes time to build up. Because of the high initial levels of SSB of the older adults who participated in this study, longitudinal studies should assess these parameters, for example, in people who have just moved into a RCFs for older adults to evaluate the evolution of the SSB. The SSB is both facilitated and constrained by the people, things, and experiences of the social environment, which dynamically interact with the individual’s character, experiences, culture, identity, and perceptions (Allen et al., 2021). In this sense, the interactions between individual and environmental characteristics make the experience of the SSB develop more or less strongly.

According to Berlin and Perone (2024), older women living in RCFs experienced a diverse range of social relationships in terms of both quality and quantity. The facility’s structure, as described by the older women in RCFs, provided an abundance of people to interact with, plus spaces and opportunities for interaction. It is likely that for the people who took part in our study, the SSB was partly fueled by their self-perceived position as members of the resident community and as individuals transcendent to the resident community, closer to the autonomy of staff. This condition of perceived in-between status helped these participants augment their SSB to the larger social environment (Berlin and Perone, 2024).

Considering this interaction, PA could facilitate this SSB since most of the participants in our study were part of a PA program. Therefore, the possibility of creating spaces for interaction among older adults using PA could increase this SSB. In the same vein, when RCFs provide quality care, sufficient activities, and stimuli, including individualized care and services and recreational support, for residents, they can improve the quality of life of older adults living in these contexts. According to Boamah et al. (2021), staff and resident relationships are particularly important and crucial to residents’ well-being and quality of life.

### 4.5 Sedentary behavior and sense of social belonging associations

The last hypothesis was confirmed as SB time was negatively associated with the SSB. Our results confirm that maintaining close and intimate social relationships in the context of the pandemic and RCFs would be negatively associated with less time spent in SB in this population (Anderson et al., 2016). The explanation of this relationship could take different ways. Social relationships could interrupt prolonged periods of SB. For example, family visits that would provoke changes of position or displacements. The residences also offer opportunities for social participation and interaction, such as meals (i.e., breakfasts, lunches and dinners). These activities modify sitting or lying positions as they generate transitions towards movement and displacement in the residences. The analysis of this result showed that the SSB could be a protective factor against social isolation (Prieto-Flores et al., 2011) and also against the feeling of loneliness in older adults living in residential care homes. Similarly, recent studies have shown that reducing these factors reduces sedentary time in older adults (Schrempft et al., 2019).

When it comes to gender, older women residing in RCFs demonstrate unique sedentary behaviors, often spending more time sitting than their male counterparts (Bellettiere et al., 2015). The most prevalent sedentary activities among these women are watching television and napping, with an average of 4.7 h a day spent on the latter (Leung et al., 2021). These two sedentary activities may limit social interactions and the SSB. As professionals in the field, we have a responsibility to address these issues. Our research suggests that older adults with a stronger SSB were more likely to engage in community programs and events, thereby reducing their sedentary time, particularly during leisure time for women (Anderson et al., 2016).

Similarly, the type of sedentary behavior should be considered when studying the sedentary behavior of older adults living in residential care facilities. Yasunaga et al. (2021) confirmed that sedentary time has positive effects if it is mentally active time (e.g., talking to others). On the contrary, the effects of mentally passive sedentary behavior on the health and well-being of older adults are adverse (e.g., watching television). In terms of context, sedentary behaviors vary by sociodemographic characteristics, including age, race/ethnicity, and education, as well as by the level of social engagement (Russell and Chase, 2019).

### 4.6 Limits

As in any longitudinal study, there was a significant loss of participants, which reduced the sample and, consequently, the generalizability of our results. In the context of the pandemic, older adults who agreed to participate in our study were subject to different restrictions and controls. A large proportion, 27 people, decided not to wear the accelerometer, which ostensibly reduced our sample. We could have elaborated on the reasons for not wanting to wear the device. The older adults may have thought it was just another control in their daily lives. Unfortunately, we could not access exchange times with the participants as there were access restrictions for our equipment during the lockdown.

Objective (accelerometers) and subjective (sense of social belonging) assessments could not be carried out simultaneously due to confinement conditions. Our team sometimes had to postpone the accelerometer evaluation for a week or even 10 days because the participants were in quarantine. Consequently, we could not respect the simultaneous control of the questionnaire and the accelerometers. This could have affected the levels of relationship between the variables. However, there were no significant differences between people who passed the questionnaires and the physical activity and sedentary behavior assessments 1 week apart.

In France, individuals’ ethnicity and racial origin could be necessary factors to study, as they may be contextual determinants of the time spent in PA and SB by older adults in RCFs. However, the French amended Data Protection Act of 6 January 1978 states that it is ‘forbidden to process personal data revealing the alleged racial or ethnic origin […] of a natural person’.

Finally, this study was only correlational, and there is a need for a study that can establish the causal relationship between levels of sedentary behavior, physical activity, and a sense of social belonging.

### 4.7 Perspectives

While moderate to vigorous levels of PA may not be the typical movement intensity for older adults, these intensities are the only ones that may reduce the effects of sarcopenia (Sánchez-Sánchez et al., 2024). As healthcare professionals and caregivers, their role in promoting PA, including these intensities, is crucial for the prevention of sarcopenia among older adults living in residential care facilities. Another perspective could be promoting strength training. It not only improves muscle strength and balance but also helps reduce fall frequency, enhances general physical condition, and decreases the fear of falling, collectively improving the quality of life and autonomy in older people (Miranda et al., 2024).

The comparison of cultural aspects related to physical activity, sedentary behavior, and sense of social belonging in older adults is an area that demands further exploration. This research is crucial as it helps us understand how these cultural aspects influence the perception of aging in residential care facilities. As Löckenhoff et al. (2009) have pointed out, European cultures and Japan, with high rates of population aging, tend to have a more negative view of aging. This is often associated with a perceived deterioration of social standing, diminished physical attractiveness, and decreased ability to perform everyday tasks and learn new things. On the other hand, cultures with younger populations, such as Malaysia, India, or China, tend to have a more positive view of aging. In France, older adults who feel they are ageist often justify it by referring to a feeling of loss: loss of respect, loss of help, and loss of social and family usefulness (Macia et al., 2007). This research highlights the need for further exploration in this area.

### 4.8 Practical implications

#### 4.8.1 Physical activity programs

Residential care facilities should implement regular physical activity programs, such as light walking sessions, soft mobility exercises and active leisure activities like gardening or dancing.

#### 4.8.2 Encouraging transitions

Encourage residents to make frequent transitions between sitting and standing positions to reduce sedentary behavior.

#### 4.8.3 Strengthening the sense of social belonging

Organize regular social activities, such as book clubs, board games, and creative workshops, to foster social interaction, strengthen the sense of social belonging, and facilitate and encourage family visits and interaction with friends to reduce social isolation.

#### 4.8.4 Environmental design

Create welcoming and comfortable communal spaces where residents can meet and socialize. In addition, residential care facilities could ensure that outdoor spaces and walking areas are easily accessible and safe to encourage outings and outdoor activities.

#### 4.8.5 Staff training

Train residential care facilities staff on the importance of light physical activities and sense of social belonging for residents’ health and well-being and encourage residents to participate actively in physical and social activities.

#### 4.8.6 Monitoring and evaluation

The residential care facilities could integrate accelerometers to objectively track residents’ physical activity and sedentary behavior time, enabling more targeted interventions and combining them with regular assessments of social sense of belonging and physical activity levels to adjust programs and interventions according to residents’ needs.

#### 4.8.7 Public health policy and practice

There could be health guidelines for residential care facilities emphasizing the reduction of sedentary behavior and the promotion of low-light physical activity and the sense of social belonging. It could be necessary to allocate resources and funding to support initiatives to increase low-light physical activity and the sense of social belonging in residential care facilities.

## 5 Conclusion

In conclusion, this longitudinal study highlights the significant associations between physical activity levels, sedentary behavior, and the sense of social belonging among older adults living in residential care facilities during the COVID-19 pandemic. The findings indicate that older adults in these settings predominantly engage in low-light physical activity and spend much time engaging in sedentary behavior. Significantly, a higher sense of social belonging is positively associated with increased physical activity and negatively associated with sedentary time. These results underscore the importance of fostering social connections and promoting low-light physical activity to enhance the well-being and health of older adults in residential care facilities. Future interventions should focus on creating environments that support social interactions and encourage regular physical activity, even at low intensities, to mitigate the adverse effects of sedentary behavior in this population. Finally, it is necessary to explore whether or not there are differences between the time spent in passive sedentary activities (e.g., watching television) and active sedentary activities (e.g., discussing with friends) since the consequences on the health and well-being of older adults living in residential care facilities are harmful in the first case and positive in the second.

## Data Availability

The raw data supporting the conclusions of this article will be made available by the authors, without undue reservation.
