# Supplementary material for: Sense of belonging and its positive association with physical activity levels and negative association with sedentary behaviors in residential aged care facilities in COVID-19 pandemic: a longitudinal study
Source: Front Psychol. 2025 Feb 5;16:1529463. doi: 10.3389/fpsyg.2025.1529463 (PMC11835940; doi:10.3389/fpsyg.2025.1529463)
Supplement: Supplementary file 1 [file Data_Sheet_1.pdf]

University and Laboratory Logo

Subject: Invitation to Participate in Daily life activities and social belonging in your residence [Residence Name].

Dear [Recipient's Name],

I am writing to you from [Our laboratory and University]. We are conducting a study on Daily life activities and social belonging in Residential Care Facilities, and we believe that your input could provide valuable insights.

Study Details:

Title: Daily life activities and social belonging in Residential Care Facilities

Purpose: We want to know your daily life activities during a typical week (during the day and while you are awake) and what is your perception of your social integration in your residence.

Participant Requirements: To achieve our objective, we need adults living in Residential Care Facilities for at least one year. You can be a man or woman over 65 years old. You live autonomously and without assistance for activities of daily living (e.g., washing, eating or shopping). If you believe you are in the cases described above, you will be able to participate in our study. To do so, we need you to wear on your hip a device called an accelerometer (the size of a watch, see picture 1, page 2) for 7 consecutive days (only when you are awake) at three times during a period of 10 months (3-month intervals). At the same time, you will have to answer a questionnaire about the perception of your residence. This questionnaire takes between 10 and 15 minutes to complete. A residence assistant will accompany you in both tasks.

Duration: The study will run from October 2020 to July 2021.

Compensation: At the end of the study, you will receive a detailed report of the overall results and, if you wish, of your individual participation. No payment will be made for your participation.

Risks and discomforts

There are certain risks and discomforts associated with this research. They include interference with electronic devices to control the pacemaker. If you are concerned by these contraindications, you cannot participate in our study.

Your participation will be useful in advancing our understanding of Physical activity and sedentary behaviours in Residential Care Facilities in France. Please let us know if you are interested in participating or if you have any questions.

Thank you for considering this opportunity to contribute to tris project.

Sincerely,

[My Name]
[My Position]
[Contact Information about research engineers]

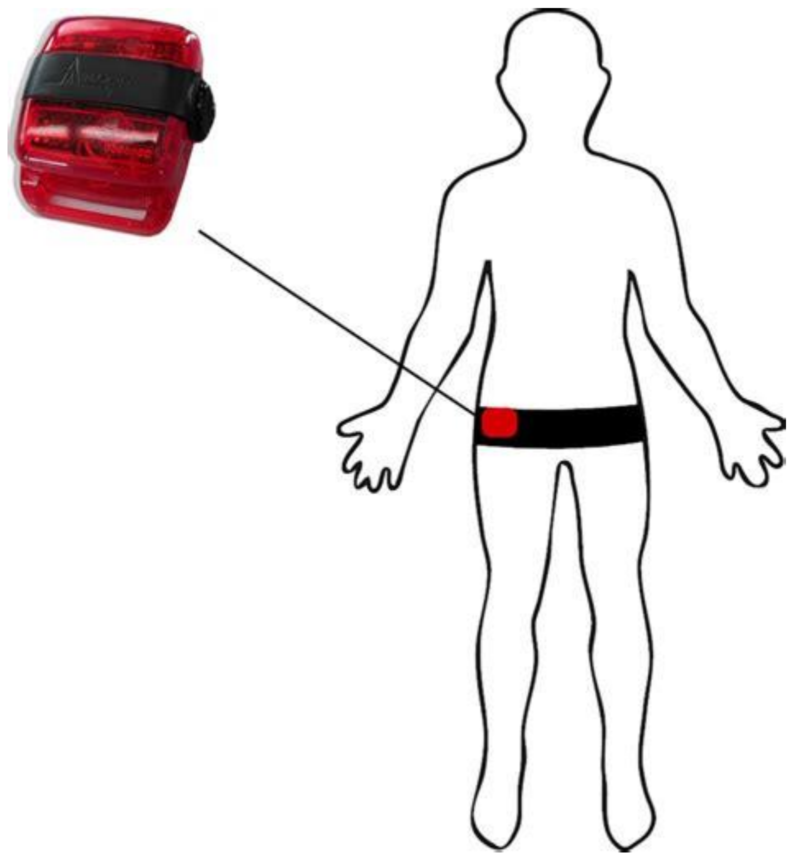

Picture 1. Accelerometer
