# Supplementary material for: Sense of belonging and its positive association with physical activity levels and negative association with sedentary behaviors in residential aged care facilities in COVID-19 pandemic: a longitudinal study
Source: Front Psychol. 2025 Feb 5;16:1529463. doi: 10.3389/fpsyg.2025.1529463 (PMC11835940; doi:10.3389/fpsyg.2025.1529463)
Supplement: Supplementary file 2 [file Data_Sheet_2.pdf]

Consent form

I, the undersigned, declare my free and informed consent to participate as a subject in the study entitled : Daily life activities and social belonging in Residential Care Facilities.

Under the direction of (research director name) :

Sponsor : University of XXXXXXXX

Principal investigator (name of research engineer) :

Purpose of study : We want to know your daily life activities during a typical week (during the day and while you are awake) and what is your perception of your social integration in your residence.
Participant's commitment: the study will consist of to wear on your hip a device called an accelerometer (the size of a watch, see picture 1, page 2) for 7 consecutive days (only when you are awake) at three times during a period of 10 months (3-month intervals). At the same time, you will have to answer a questionnaire about the perception of your social integration to your residence.

Principal investigator's commitment: as principal investigator, I undertake to conduct this research in accordance with ethical and deontological provisions, to protect the physical, psychological and social integrity of individuals throughout the research, and to ensure the confidentiality of information collected. I also undertake to provide participants with all the support they need to mitigate any negative effects that may arise from participation in this research.

Participant freedom: consent to continue the research may be withdrawn at any time without giving any reason and without incurring any liability or consequence. Answers to questions are optional, and failure to answer will have no consequences for the subject.

Participant information: the participant may obtain additional information about this study from the principal investigator, within the constraints of the research plan.

Confidentiality of information: all information concerning participants will be kept anonymous and confidential. Data processing is not nominative, and is therefore not covered by the French Data Protection Act (the right of access and rectification is not admissible). As this research is only psychological in nature, it is not covered by the Huriet-Sérusclat law on the protection of individuals in biomedical research. The transmission of information concerning the participant for expert appraisal or scientific publication will also be anonymous.

Deontology and ethics: the promoter and principal investigator undertake to maintain absolute confidentiality and professional secrecy with regard to all information concerning the participant (Title I, articles 1, 3, 5 and 6 and Title II, articles 3, 9 and 20 of the Code of Ethics of Psychologists, France).

Date and place : Done at on in 2 copies

Signatures :

The participant The principal investigator
