# Supplementary figures and images for: Sense of belonging and its positive association with physical activity levels and negative association with sedentary behaviors in residential aged care facilities in COVID-19 pandemic: a longitudinal study
Source: Front Psychol. 2025 Feb 5;16:1529463. doi: 10.3389/fpsyg.2025.1529463 (PMC11835940; doi:10.3389/fpsyg.2025.1529463)

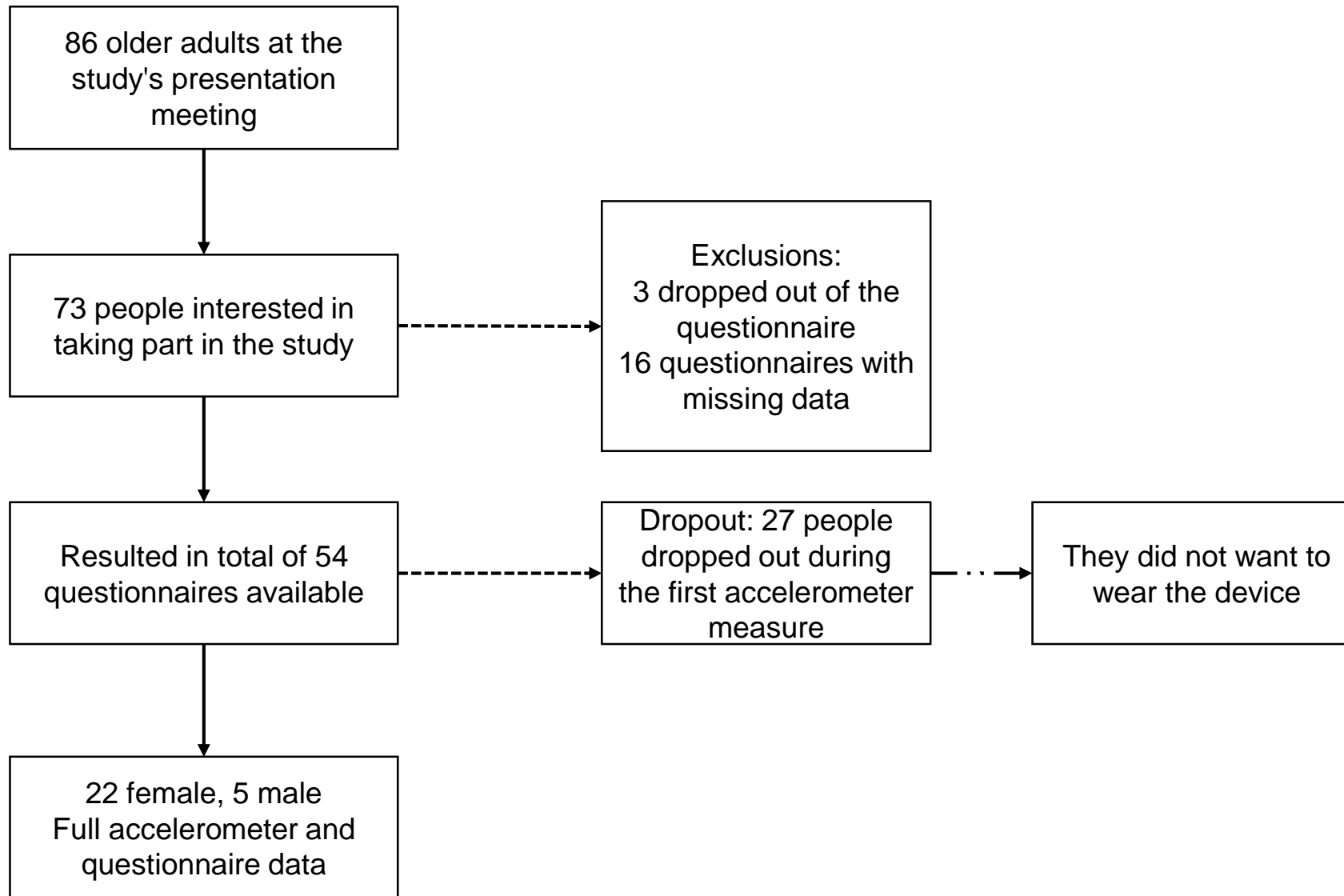

Supplemental material 3. Flowchart of the study

Supplement: Supplementary file 3 [file Data_Sheet_3.pdf]
